# Comparison of fresh and aged lithium iron phosphate cathodes using a tailored electrochemical strain microscopy technique

**DOI:** 10.3762/bjnano.11.46

**Published:** 2020-04-07

**Authors:** Matthias Simolka, Hanno Kaess, Kaspar Andreas Friedrich

**Affiliations:** 1 University of Applied Sciences Esslingen, Faculty of Basic Sciences, 73728 Esslingen, Germany; 2 German Aerospace Center (DLR), Institute of Engineering Thermodynamics, Pfaffenwaldring 38–40, 70569 Stuttgart, Germany; 3 University of Stuttgart, Institute of Building Energetics, Thermal Engineering and Energy Storage (IGTE), Pfaffenwaldring 31, 70569 Stuttgart, Germany

**Keywords:** activity, ageing, cathode, electrochemical strain microscopy (ESM), LiFePO_4_

## Abstract

Electrochemical strain microscopy (ESM) is a powerful atomic force microscopy (AFM) mode for the investigation of ion dynamics and activities in energy storage materials. Here we compare the changes in commercial LiFePO_4_ cathodes due to ageing and its influence on the measured ESM signal. Additionally, the ESM signal dynamics are analysed to generate characteristic time constants of the diffusion process, induced by a dc-voltage pulse, which changes the ionic concentration in the material volume under the AFM tip. The ageing of the cathode is found to be governed by a decrease of the electrochemical activity and the loss of available lithium for cycling, which can be stored in the cathode.

## Introduction

The growing demand for safe, reliable and efficient energy storage is supporting the development and improvement of current battery technology. Since the introduction of the first Li-ion battery by Sony in the 1990s, the energy and power density have increased yearly and commercial cells are much safer now as compared to their first entrance to the market. However, especially for the automotive sector, the current battery capabilities are still inferior with respect to the expectations of many users regarding energy density and recharging time. Furthermore, recent life-cycle analysis (LCA) studies have emphasized the issues associated with battery production and recycling [1–3]. As a consequence there is a trend to reduce or eliminate cobalt as a critical raw material [4–5]. Lithium iron phosphate (LiFePO_4_ or LFP) is highly promising to achieve this goal but further improvements are necessary. These improvements are not only focused on higher energy and power density, but also the need to offer a longer product life with a stable capacity and power capability.

For the analysis of ageing mechanisms, a variety of techniques are available. In the category of destructive and post-mortem methods, scanning electron microscopy (SEM) is widely applied, due to the deep insights into the material that it can provide, e.g., insights related to structural changes and generated surface layers. In combination with X-ray techniques like energy dispersive spectroscopy (EDS or EDX) it adds chemical information on the elemental distribution to the structural analysis. Further methods that have been applied to study ageing in LFP are X-ray photoelectron spectroscopy (XPS), inductively coupled plasma (ICP), transmission electron microscopy (TEM), focused ion beam (FIB) SEM and X-ray absorption near edge structure (XANES) [6–13]. Another technique for post-mortem analysis is atomic force microscopy (AFM). In its basic form, it provides information on the topography of the sample. More advanced AFM modes extract in addition to the topography additional mechanical (stiffness, elasticity), electrical (conductivity, surface potential), electrochemical (reactivity, mobility and activity), mechanoelectrical (piezoelectricity) and chemical (chemical bonding) material properties. In situ AFM imaging of the sample topography is often used to study the solid electrolyte interface (SEI) on graphite anodes and HOPG [14–16], Li metal [17] and on cathode materials [18–19] as well as the changes in particle size during ageing [19–20]. Other AFM modes used for the analysis of ageing are, for example, Kelvin probe force microscopy (KPFM) and conductive AFM (CAFM). Luchkin et al. used KPFM to analyse the Li-ion distribution in graphite anodes and found a core–shell structure in aged graphite particles [21]. Wu et al. used KPFM to track the changes in the surface potential of LiCoO_2_ cathodes during ageing and found a decrease of the surface potential with ageing, due to irreversible phase transitions, side reactions on the surface and coarsening of grains [22]. Hiesgen et al. used CAFM to study the degradation of lithium–sulphur cathodes during ageing and found a strong decrease of the conductive area of the sample, which correlated well with the capacity degradation of the samples [23].

Electrochemical strain microscopy (ESM) is a relatively new AFM contact mode, which probes ionic charges accumulated in a small volume under the AFM tip after application of an electric field by measuring the resulting surface strain [24–26]. It was first experimentally introduced by Balke et al. [24–25]. Morozovksa et al. [26–28] provided the theoretical background. If an alternating electric field is applied, the ions located in the sample volume under the AFM tip are forced to oscillate, which generates strain due to concentration-dependent Vegard expansion. The strain finally creates an oscillating surface displacement with material specific amplitude, which is measured by the AFM tip. Using an additional dc-voltage pulse in the millisecond range, the concentration in the volume under the AFM tip can be altered even further. The ac-voltage with the resulting oscillating surface displacement amplitude (ESM signal) is then employed to track the induced concentration change due to the dc-voltage pulse – this technique is called time-domain ESM. In the simple approximation, given in Equation 1, the resulting surface displacement *u*^S^ is proportional to the product of the Vegard expansion coefficient of the host material β^H^ and the change in ionic concentration of the ionic component δ*c*^Li^, in our case Li [26,29].

[1] $$u^{\text{S}}\propto \beta^{\text{H}}\delta c^{\text{Li}}$$

The drawback of the ESM technique is the possible additional contributions to the ESM signal from other mechanisms. These mechanisms are piezoelectricity, flexoelectricity and electrostriction. Further contributions are possible by deformation potential generation, electron–hole formation, coupling of electrons and phonons, electrochemical side reactions in the tip–sample junction, electrostatic interaction, and volume expansion related to temperature changes and charge injection from the tip [27,29–34]. However, the current state of research assumes the Vegard expansion to be the main signal generation mechanism. The other influences are expected to play only a minor role, since they either have a small signal generation magnitude compared to the Vegard expansion or their time scales are much shorter than the relaxation times for ESM experiments (Maxwell relaxation times) [30,32]. Electrochemical side reactions may create surface features, which, however, are detectable by subsequent scans of the same location. Additionally, since in a dry argon atmosphere no water meniscus is available to serve as electrolyte, side reactions are avoided. Further information of the non-Vegard expansion related influences is given in the supporting information in [34]. It is possible that the ESM signal is not solely generated by one mechanism, but the sum of several contributions (as it is as well assumed by Kalinin and Morozovska [32], with the Vegard contribution being the predominant). However, the variation of the ESM signal after the dc-voltage pulse is governed by ionic movements and reflects therefore the diffusivity of Li-ions in the material [29–30,33,35–36]. The basic fact that the ESM signal itself is dependent on the presence of ionic species was shown by Schön et al. [37] and Sasano et al. [38–39], who combined EDX, electron backscatter diffraction (EBSD) and ESM measurements. Therefore, even with the assumption that the Vegard expansion is not the major contribution to the measured ESM signal, its origin is the ionic movement within the material and hence can be used for the ageing analysis and diffusivity analysis. A further discussion regarding non-Vegard related contributions on the ESM signal can be found in Supporting Information File 1.

Since the variation of the ESM signal is governed by migration and diffusion processes, it can be used to fit relaxation functions and extract the characteristic time constant τ. Diffusion processes are often fitted using an exponential decay function [30,33,36,40], but the power law [29] or stretched exponential decay [41] functions are applied, too. The drawback of fits employing single exponential functions is the poor fitting quality compared to higher-order exponential functions or fitting functions with more parameters. Nevertheless, the additional fitting parameters increase the complexity of the interpretation and can lead to false conclusions [33,36]. Therefore, we used a single exponential decay function in form of Equation 2 for the fitting of the measurement signal with *b* = 1/τ.

[2] c(t)=aexp(−bt)+c

Luchkin et al. used ESM to study the changes in fresh and aged LiMnO_2_ cathodes and found a decrease of the diffusion coefficient in the aged sample due to structural degradation of the material [30]. Zhu et al. studied the degradation of LiNi_0.3_Co_0.3_Mn_0.3_O_2_ by ESM and showed a decrease in the ESM amplitude over the ageing of the cathode material, which they attributed to a reduced electrochemical activity [42]. Yang et al. report similar findings; they used ESM to study thin film Li_1.2_Co_0.13_Ni_0.13_Mn_0.54_O_2_ cathodes and observed decreasing ESM amplitude after a few ESM scans. They attributed this, similar to Zhu et al., to the reduction of the electrochemical activity, electrochemical fatigue and degradation of the material [43]. Jesse et al. conducted ESM on thin film silicon anodes and aged them by high frequency cycling [33]. Contrary to Yang et al. and Zhu et al., they found an increase of the ESM amplitude over cycling time, which they linked to an increase in electrochemical activity and higher lithium concentration in the anode. In general, the ionic mobility, concentration and activity in the probed volume and the material structure influence the ESM signal. Several groups already investigated LFP by ESM measurements. Chen et al. studied the influence of the material properties and preparation of fresh LFP samples on the ESM signal and showed that the material structure influences the electrochemical activity [44–45]. Eshghinejad et al. used LFP for the validation of their theoretical and modelling framework and demonstrated the correlation between ionic concentration and diffusivity and the ESM signal [46]. In these studies, the ESM measurements were performed on fresh samples and the degradation due to cycling was not considered. Degradation analysis of cathode materials using ESM has been performed before, for example for LiMnO_2_ [29–30] and nickel manganese cobalt oxide (NMC) [42–43] but, to the best of our knowledge, not for LFP.

In this paper, we analyse the electrochemical activity and Li-ion concentration using the ESM technique and show a decrease of the electrochemical activity and a reduced active Li content in the aged cathode due to the cycling. The reduction of the electrochemical activity and the reduced Li content in the aged cathode explain the capacity loss of the commercial cell, since active material turns electrochemically inactive and is therefore lost for cycling. At the same time, electrochemically inactive material traps Li-ions, which reduces the remaining capacity of the cell. Therefore, the presented ESM measurements visualize the reduction of the electrochemical activity and the loss of lithium inventory of the sample due to ageing on the nanometer scale. With that, ESM offers a higher resolution technique for the visualization of the activity compared to digital volume correlation [47–48] and digital image correlation [49], which have a resolution of 2 µm or more.

## Experimental

### Cell ageing and sample preparation

The cells are commercial 26650 LiFePO_4_ cells from A123 Systems LLC with a nominal capacity of 2.5 Ah and a voltage window from 2.0 to 3.6 V. A group of cells was cycled at +55 °C using a part from the worldwide harmonized light vehicles test procedure (WLTP) driving profile and one cell was used for further analysis in comparison with a fresh cathode. After the cell reached a total discharge capacity of 2000 Ah, it had a remaining discharge capacity at 1C of 2.156 Ah, which represents a capacity loss of 17% with respect to its original value. The discharge curves are shown in Supporting Information File 1, Figure S1. The cells were disassembled inside a glovebox under argon (MBraun, O_2_ and H_2_O < 2 ppm) and washed with dimethylcarbonate (DMC, Sigma-Aldrich). Cross-section cuts were obtained with an unfocused argon beam cross-section polisher (Jeol, 19520-CCP). The transfer of samples was done inside a transfer vessel to avoid any contact with air.

### ESM measurements

ESM analysis was conducted with a Bruker Icon instrument inside a glovebox (MBraun, O_2_ and H_2_O < 2 ppm), equipped with a Zurich Instruments lock-in amplifier (HF2LI), a signal access module (SAM V) and PeakForce quantitative nanomechanical properties (QNM) module. In addition to the ESM signal, the topography of the sample and the deflection error of the AFM tip are recorded. The deflection error represents the feedback signal of the feedback control system for the tip–sample contact force control and is the difference between the set point and the effective value. The ESM signal is based on the real, in-phase amplitude X from the lock-in, because, following the theory of Morozovska et al. [26,50], it is the main signal part carrying the ESM signal. This was already shown in previous measurements, for which the signal intensity of the real, in-phase amplitude (X) has the same magnitude as the absolute signal amplitude (R) [34]. For data acquisition, we use a National Instruments card (PCI-6111) on a separate computer controlled by a LabVIEW (R2016) routine. Electric conductive commercial tips from NT-MDT, coated with W_2_C with a spring constant of about 3.5 N/m and a resonance frequency of about 77 kHz, are used (HA_FM/W2C+). Tip calibration was performed by the thermal tuning of the AFM with the adaption of the deflection sensitivity. The measurements have a pixel density of 256 × 100 measurement points, which means that the pixels have a rectangular shape in the direction of the scan direction (left to right) and are not squares. ESM measurements are done with the AFM interleave mode in contact with the sample surface. The interleave mode scans the same line twice, once as the main scan to record standard AFM signals and a second time during which the voltage profile is applied to the tip and the vertical tip deflection is recorded. Each point consists of a measurement period with a dc-voltage pulse of |3| V if not otherwise stated for 10 ms followed by a dc-voltage off period of 15 ms. The |3| V dc-voltage amplitude was chosen as a compromise to minimise tip wear while keeping a distinct signal quality at the same time. An ac-voltage with 40 kHz and if not stated otherwise with a 2 V amplitude is overlaid over the dc-voltage during the whole measurement time. The ESM technique applied here does not use any resonance enhancement to amplify the ESM signal (such as dual resonance frequency tracking (DRFT or DART) or band excitation (BE)), but uses a single tracking frequency, as it was already performed by Luchkin et al. [30]. Using a single tracking frequency far off the resonance frequency range of the tip–sample system limits the measurable signal intensity, but avoids any measurement errors, which can arise due to tracking errors of the shifting resonance frequency [31]. The dc-voltage generates a concentration change in the sample volume under the tip and the ac-voltage is applied to follow the changes in ionic concentration by tracking the amplitude of the surface oscillation, which is induced by the ionic vibration in the material. Due to the modified experimental set-up, the presented ESM technique does not correspond to the classical ESM technique introduced by Balke et al. [24], but is similar to the time-domain measurements reported by Jesse et al. [33], therefore we call it tailored ESM. The application of the interleave contact mode offers the opportunity to record the sample surface independently from any applied voltage during the first scan line and a second time during the interleave contact mode with the applied voltage profile. Therefore, any surfaces changes, which are generated due to the applied voltage, are easily observed by comparing the topography of the first (standard) and second (interleave contact mode) scan. Nevertheless, the theoretical background is still based on the comprehensive publications by Morozovska et al. [26–27,50–51]. Further information about the set-up with control experiments regarding the origin of the signal can be found in [34]. The ESM measurements were performed on micrometre-sized single particles of a cross-section of the electrodes cut as specified above.

## Results and Discussion

### Cell and cathode characterization

**Figure 1:**
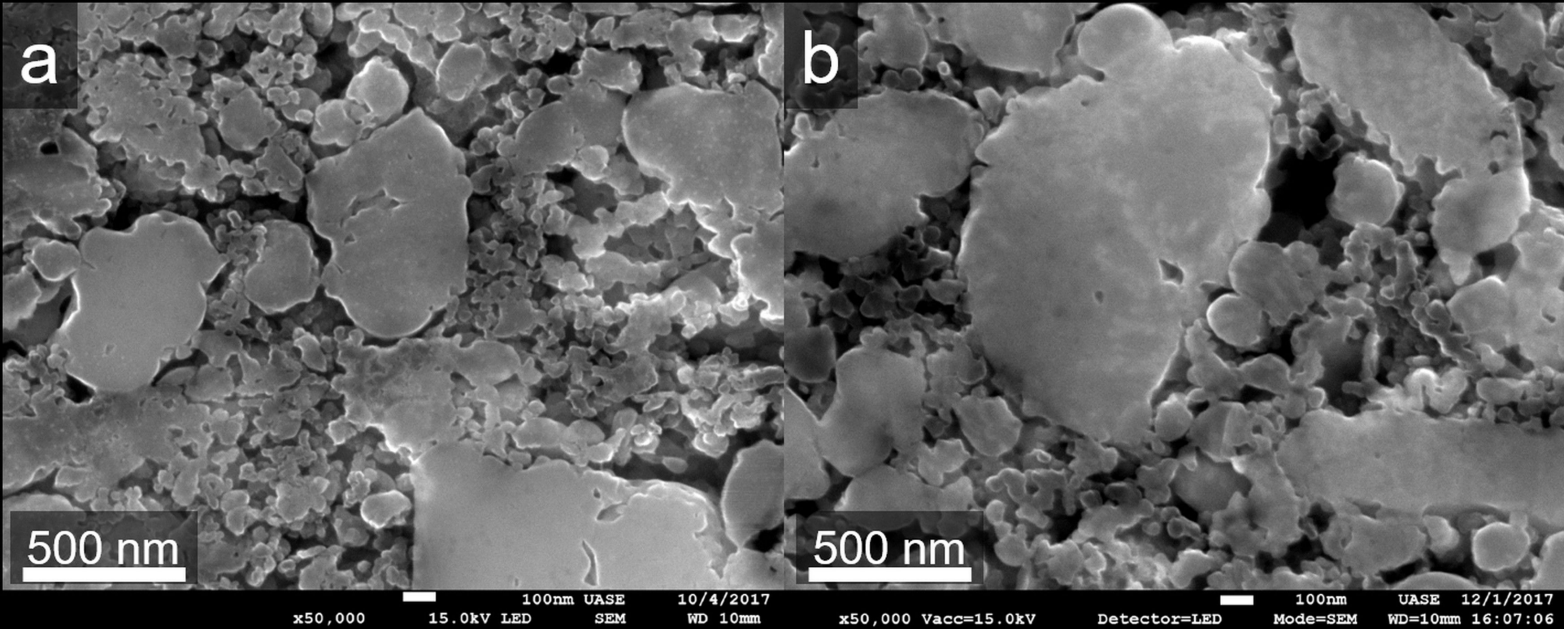
SEM cross-section view of the fresh (a) and aged (b) cathode.

The ESM analysis was conducted inside of particles of the cross-sections of the fresh and aged cathodes. Two examples of the cross-section structure of the cathodes are given in Figure 1. In Figure 1a the fresh and in 1b the aged cathode cross-section is shown. The electrode consists of particles ranging from 50 nm to a few micrometres in diameter. No evident differences can be discerned on this scale when comparing pristine and aged samples. The voltage over discharge capacity plot of the commercial full cells is shown in Supporting Information File 1, Figure S1. It displays the capacity loss of the aged full cell after cycling. Due to the anode contribution to the capacity loss in the commercial full cell setup, the cathode was additionally analysed separately. The cathode ageing is observed in the Nyquist plot in Figure S2 from the fresh and aged cathode vs lithium metal reference electrode in a three-electrode test cell. More information about the experimental setup is given in Supporting Information File 1. Following the approach proposed in the literature, the first semi-circle at high frequencies is assigned to the cathode and the second semi-circle at mid frequencies to the lithium anode [52–53]. The aged sample exhibits a larger first semi-circle due to ageing and the second semi-circle stays nearly constant, since the lithium reference anode is not affected by the cycling. In Figure S3, the fresh and aged cathode are cycled in a three-electrode setup combined with a fresh anode for both cathodes. Looking at the first charging step, the aged cathode exhibits a smaller charge capacity compared to the fresh cathode. This indicates a smaller amount of lithium stored or available for the electrochemical process or a reduced amount of electrochemically active cathode material. The capacity loss from first charge to first discharge is attributed to surface layer generation (anode: solid electrolyte interface, SEI; cathode: solid permeable interface, SPI) on both electrodes, since they were rinsed before the full-cell assembly. After the first cycle, the capacity stays constant (not shown here). The discharging capacity is higher for the fresh cathode compared to the aged.

### Analysis of the ESM signal of the fresh cathode cross-section

**Figure 2:**
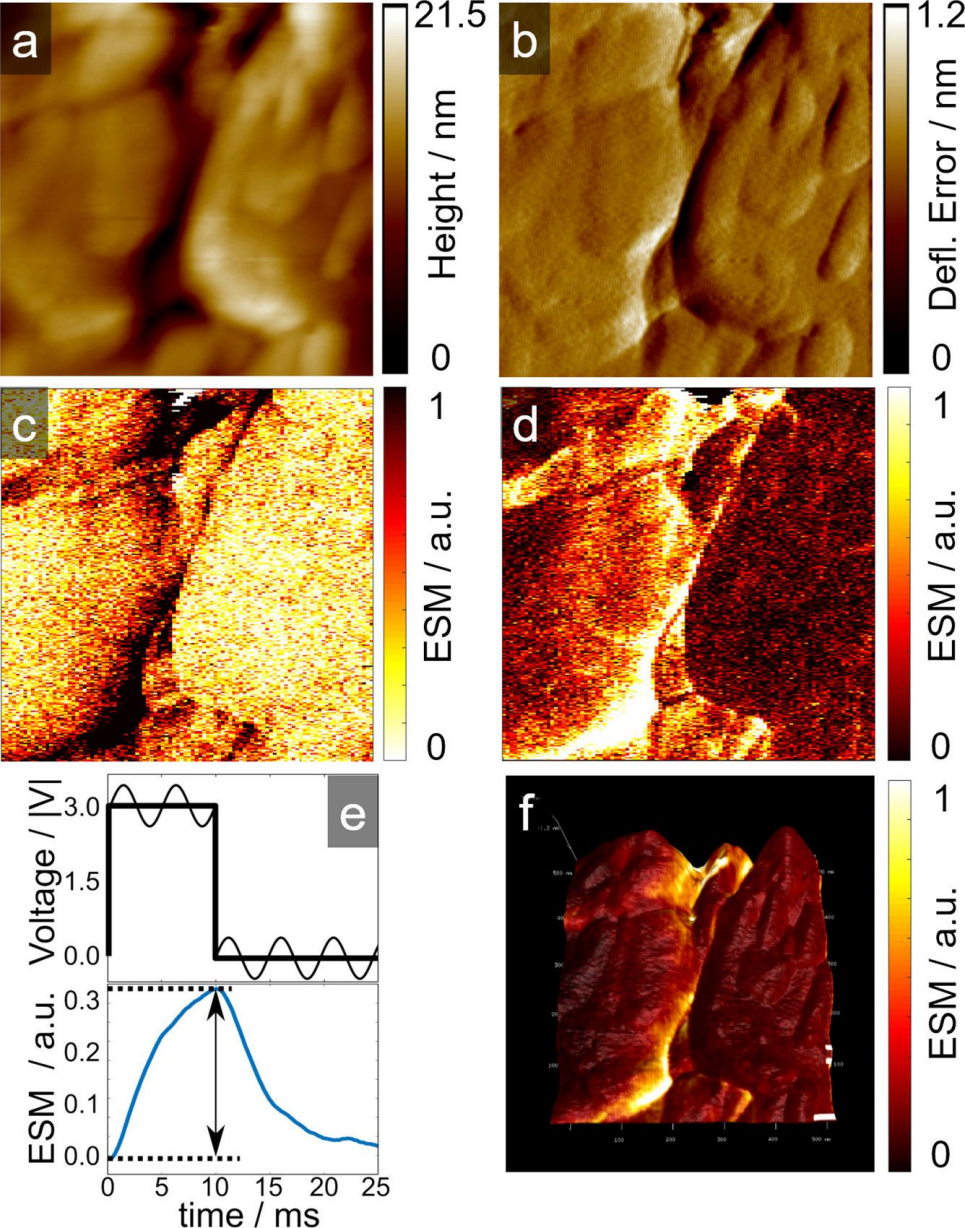
Topography (a), deflection error (b) and ESM amplitude due to the positive and negative voltage pulse (c and d) at the fresh cathode. In e), an example of the measured ESM signal with the applied voltage pulse are shown. In f) is the result from d) overlaid on the topography in a). Scan size is 1 × 1 µm^2^.

The ESM measurement in Figure 2 displays differences of the ESM signal within one particle. In Figure 2a and 2b, the topography and the deflection error of the particle and in Figure 2c and 2d the ESM signal due to the application of a positive and negative voltage pulse are shown. Figure 2e displays the applied voltage pulse with the dc and ac-voltage part at the top and the resulting ESM signal at the bottom, which is generated due to the increase or decrease of the ionic concentration in the probed volume due to the electric field. During accumulation of Li-ions with the dc-voltage pulse, due to the electric field driven migration, the ESM signal increases. Afterwards, when the dc-voltage is turned off, the ESM signal decreases due to the concentration driven diffusion and the decrease of the ionic concentration in the probed volume. The difference between the signal intensities at the beginning and at the end of the dc-voltage pulse is extracted (indicated by the arrow in the bottom part) and represents one pixel in the 2D presentation of the ESM data.

The ESM signal, which we observe on carbon-coated LFP, is not only limited to structural boundaries within one single grain, which are known to exhibit a high mobility for ions due to structural disordering, high concentration of defects and lower energy barrier [40,54–55]. We also observe a homogeneous high signal on planar locations inside the particle. The variation of the ESM signal inside the particle is most likely caused by the anisotropic ionic mobility of the olivine structure of LFP. The olivine structure exhibits preferential lithium-ion transport along the [10] channel of the lattice [56–57], which therefore influences the ESM signal intensity, since the preferential lithium-ion transport direction induces a high concentration change during the dc-voltage pulse, while for ionic blocking directions, no concentration change is achieved and therefore no change in the ESM signal is produced. The olivine crystal structure on the planar locations is not the only influence on the ionic mobility, but it is an additional factor next to the structural disordering, high concentration of defects and lower energy barrier and more important on planar locations than on structural boundaries.

We can exclude any significant influences due to changes of the tip–sample contact or edge artefacts in the ESM signal, since trace and retrace, or positive and negative dc-voltage pulse respectively, show similar signals for the sample locations. If tip–sample artefacts and increase of the tip–sample contact area would influence the signal generation, the collected signals from trace and retrace would show locations with different response for trace and retrace and a direction dependence for the tip movement. In Figure 2f, the ESM signal from Figure 2d is overlaid on the topography from Figure 2a. It indicates that the signal is not influenced by the topography of the sample, since the flat locations in the top and bottom of the image show a distinct ESM signal. Additionally, due to the slow scan speed of 0.2 Hz, we assume a stable tip–sample contact during the measurement. Considering that the measurements are conducted on cross-sections of particles, we neglect the carbon coating or binder material influence on the results. The ESM signals show some precise and fine structures and clear separations within the particles. Assuming a rather large probed volume of the cubic tip radius (*R*_tip_ around 30 nm or even larger), the ESM signal would exhibit rather diffuse signal allocation. Therefore, it is more reasonable to assume a limited probed volume close to the surface of the tip–sample junction of only a few nanometres in depth.

### Dependency of the ESM signal intensity during stepwise increase of the dc-voltage amplitude

**Figure 3:**
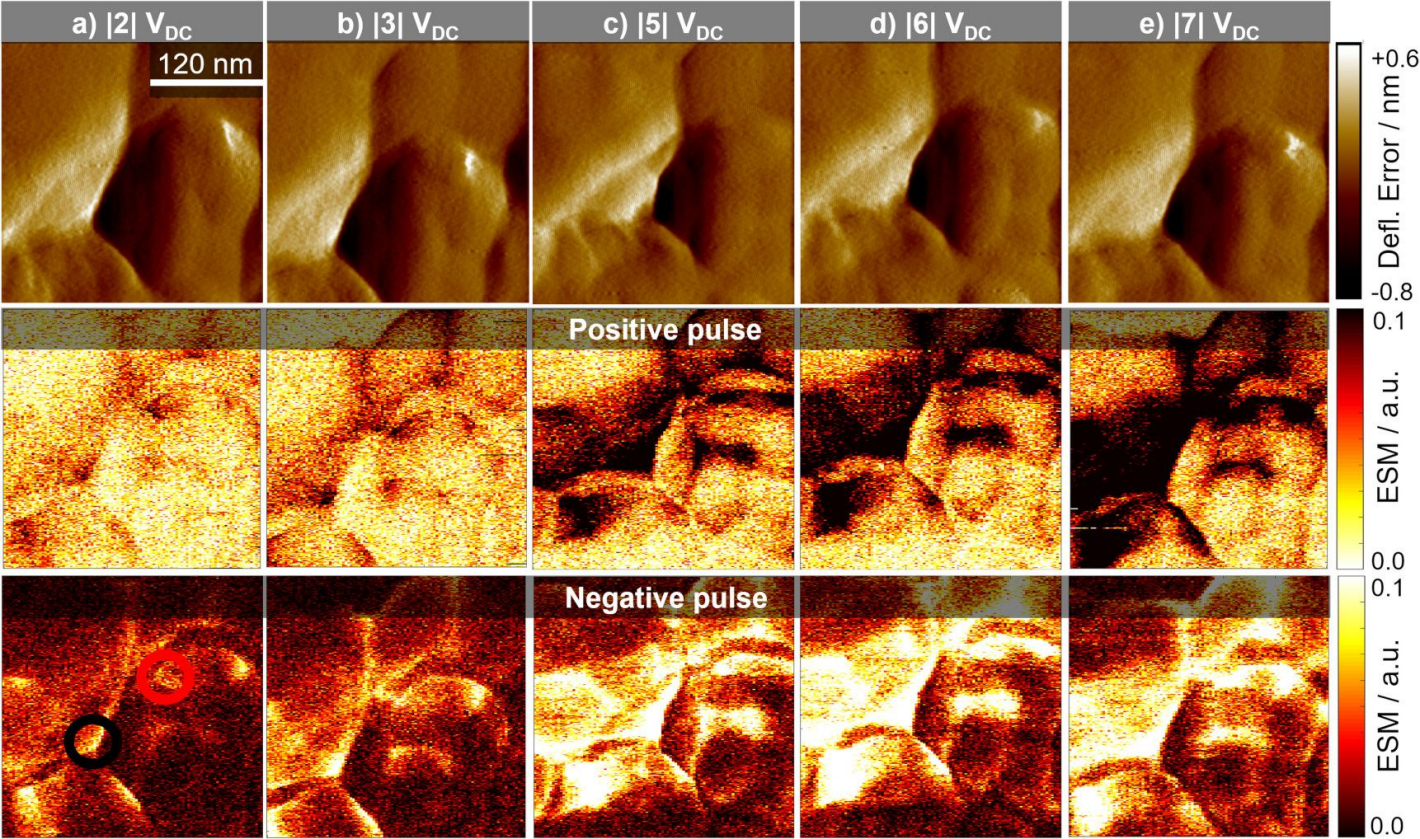
Comparison of different dc-voltage amplitudes at the same location of a fresh cathode. The top row shows the deflection error, the middle row the ESM signal during positive dc-pulse and the bottom row during negative dc-voltage pulse. In a) with |2| V, b) with |3| V, c) with |5| V, d) with |6| V and e) with |7| V. Scan size is 0.33 × 0.33 µm^2^.

**Figure 4:**
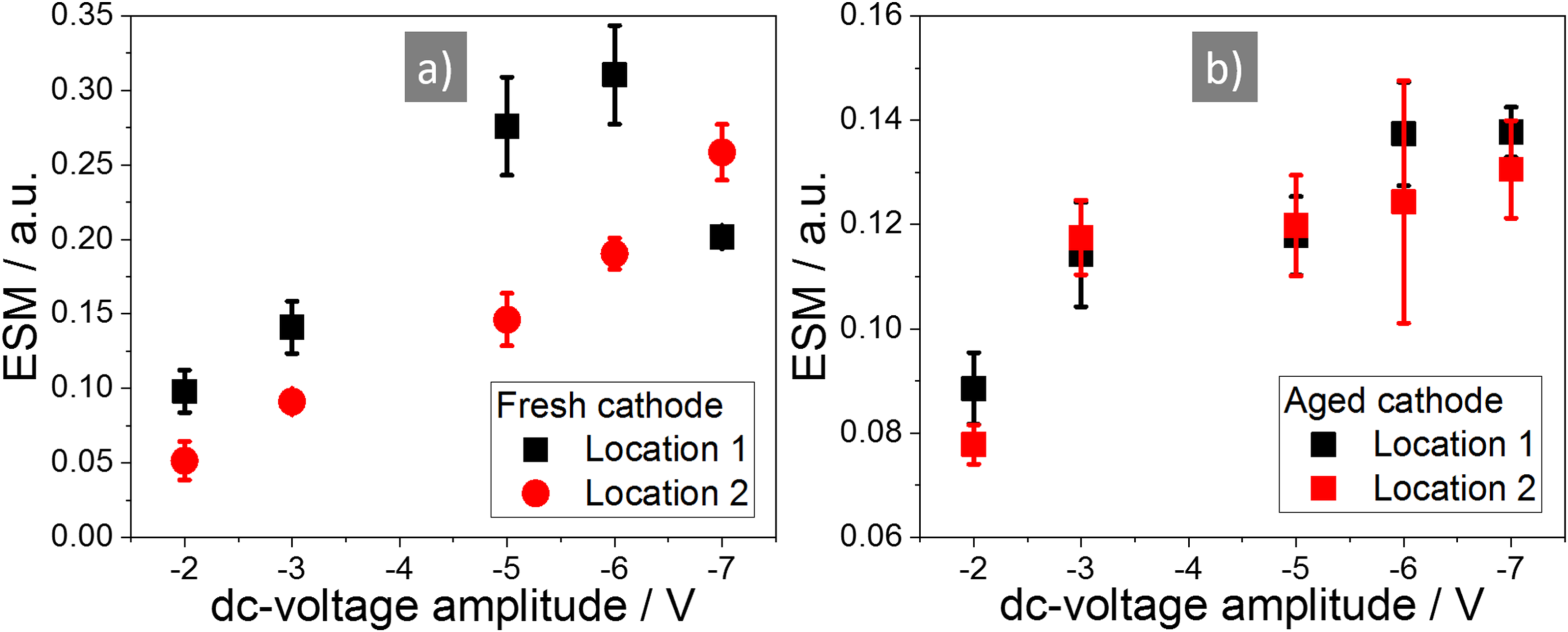
Evolution of the ESM signal intensity at the a) fresh cathode and b) aged cathode with the stepwise increasing dc-voltage pulse. The locations for the fresh cathode are marked in Figure 3a in the bottom row. For the aged cathode see Supporting Information File 1, Figure S5.

To analyse the dependency of the ESM signal on the applied dc-voltage amplitude, the same location was measured repeatedly with a stepwise increased dc-voltage amplitude after each measurement. The results for the different dc-voltage amplitudes are displayed in Figure 3 with a) |2| V, b) |3| V, c) |5| V, d) |6| V and |7| V dc-voltage amplitude in e). The middle row shows the ESM signal due to the positive and the bottom row due to the negative voltage pulse. With increasing dc-voltage amplitude, the overall active area of the sample increases, as can be seen by the increasing fraction of the sample showing a distinct ESM signal. At lower dc-voltage amplitudes (|2| V and |3| V), there are mainly structural boundaries visible in the ESM signal. At |3| V, areas with roughly 50 nm diameter are visible. Stepping up the dc-voltage amplitude further increases the overall active area. Still, some locations in the sample stay inactive, even at a dc-voltage of |7| V. Contrary to other publications, irreversible changes, or the generation of surface features at higher voltage amplitudes (|5| V to |7| V), are not observed [43,58]. This is probably due to the smaller excitation dc-voltage amplitudes, the short excitation time of only 10 ms and the inert gas atmosphere. This prevents the generation of a water droplet meniscus at the tip–sample junction, which can serve as an electrolyte for electrochemical reactions. The increase of the ESM signal intensity for the fresh cathode is shown in Figure 4a. It exhibits a linear increase of the ESM signal with increasing dc-voltage amplitude, which is in agreement with theoretical work done by Morozovska et al. [26]. Only at location 1, at −7 V dc-voltage amplitude, the signal intensity decreases as compared to −5 V and −6 V. This indicates either an irreversible change in the ionic concentration in the probed volume due to the preceding measurements, or a degradation of the material structure due to the applied electric field. Since the ESM signal depends on the change in ionic concentration during the applied dc-voltage pulse, an irreversible accumulation of Li-ions in the probed volume due to the preceding measurements would decrease the feasible change in concentration and therefore could reduce the ESM signal intensity. Similar, structural degradation could influence the ESM signal intensity by the reduction of the ionic conductivity. Yang et al. observed a similar decrease of the ESM signal intensity after scanning the same location several times, which they attributed to either changing ionic concentration or degradation of the electrochemical activity [43]. The second location shows a linear increase of the ESM signal intensity over the whole range of the applied dc-voltage amplitudes, but with a smaller slope compared to location 1 and ESM signal intensity. The measurements in Figure 3 show a clear dependency of the ESM signal intensity on the excitation voltage. The inactive locations – to be precise: the locations, which do not generate any ESM signal in the sample – stay inactive and cannot be activated by an increasing dc-voltage amplitude, at least for the dc-voltages applied here. Structural boundaries in the particle result in general higher ESM signal intensity compared to homogeneous and planar locations, which points towards the importance of nanostructuring of battery materials to increase the boundary density. Moreover, the results from Figure 3 indicate that by using a |3| V dc-voltage amplitude for further measurements, the limiting factor for the resulting ESM signal intensity is not the concentration limit in the probed volume, but the mobility and activity of the Li-ions, which is influenced by structural aspects.

### Comparison of the ESM signal on fresh and aged cathodes

**Figure 5:**
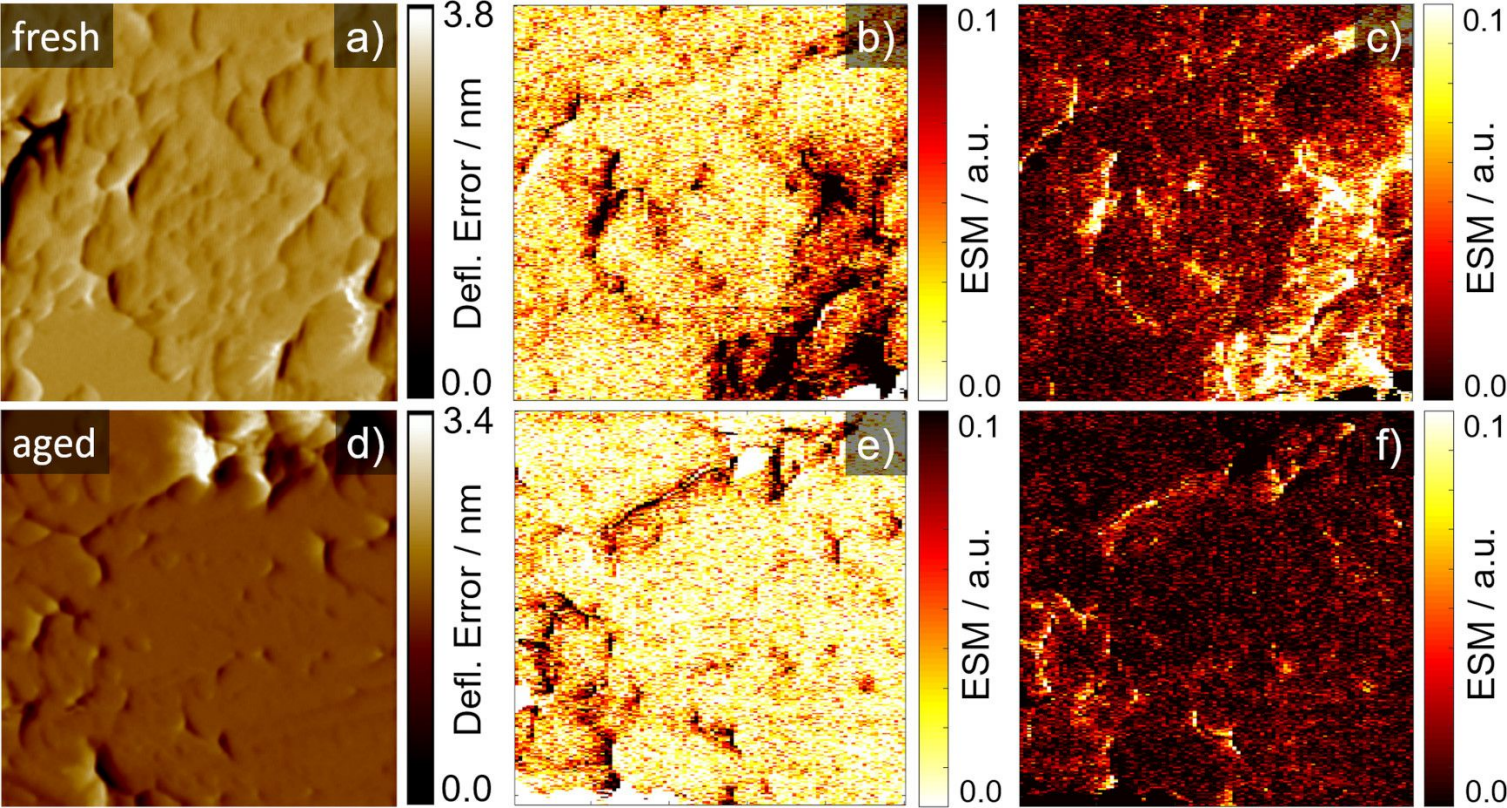
ESM measurements of a fresh (top row) and aged cathode (bottom row) cross-section. a) and d) show the deflection error, b) and e) ESM signal during positive voltage pulse and c) and f) during negative voltage pulse. Scan size is 1 × 1 µm^2^.

**Figure 6:**
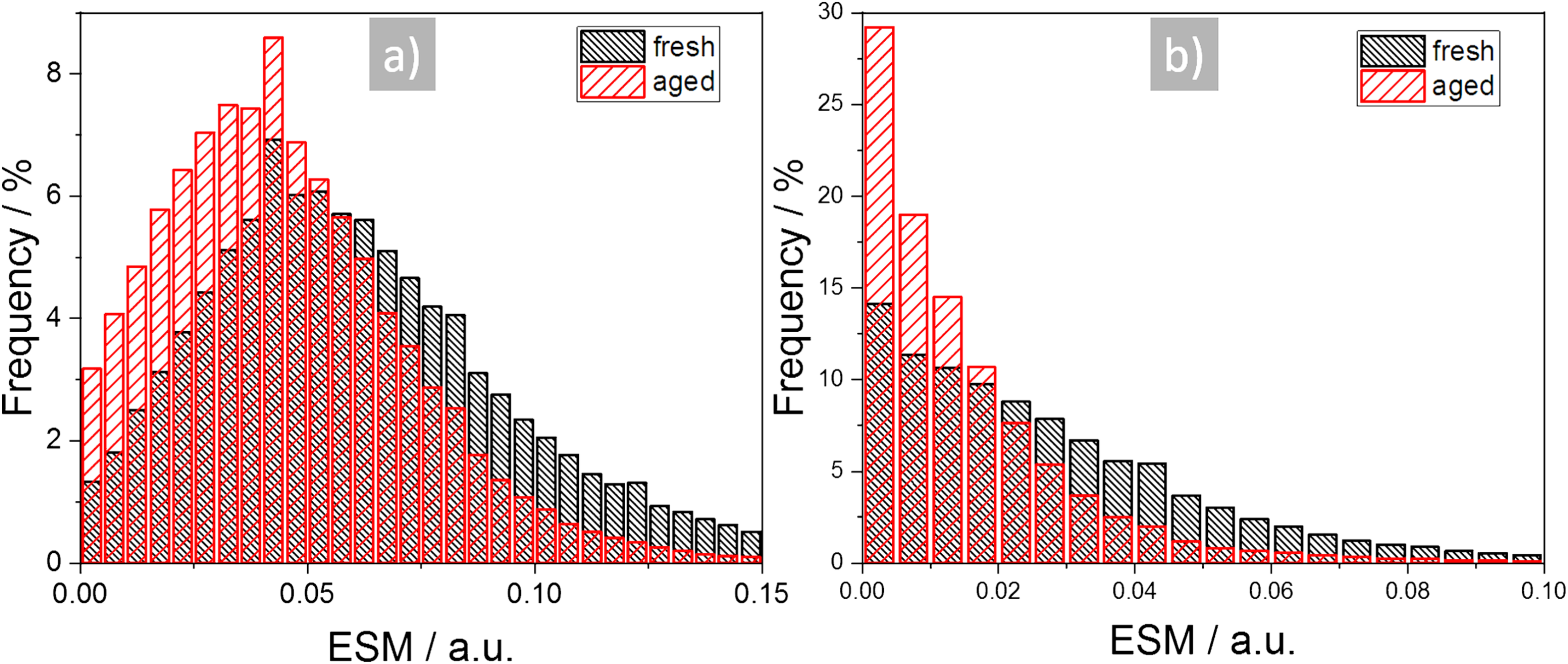
Comparison of ESM signal intensity of the fresh and aged cathode due to the a) positive and b) negative dc-voltage pulse.

Ageing of battery material is a complex process with different mechanisms happening simultaneously and influencing each other. Here we analyse the change of the ESM signal over ageing by comparing the fresh cathode with an aged cathode sample. Figure 5 compares the ESM signal of the fresh (a–c) and aged (d–f) sample with the deflection error (a and d), the ESM signal due to the positive (b and e) and negative (c and f) dc-voltage pulse. In the fresh sample, structural boundaries and homogeneous planar locations show a distinct ESM signal. In the aged sample, only structural boundaries possess a distinct ESM signal. Large parts of the measured area are inactive and exhibit nearly no ESM signal at all. Additionally, the ESM signal intensity is smaller compared to the fresh sample, at least for voltages higher than |5| V. The voltages at |2| and |3| V show comparable ESM signal intensities at the analysed locations for the fresh and aged sample. However, as it is shown in Figure 6 and the following discussion, the overall ESM signal intensity decreases in the aged compared to the fresh sample already with a voltage amplitude of |3| V. This indicates that in the aged sample with the same magnitude of the electric field, only a smaller degree of concentration change at fewer locations in the sample is generated. As can be seen in the stepwise increase of the dc-voltage pulse amplitude in Figure 4b and Supporting Information File 1, Figure S5, especially with higher dc-voltage amplitudes only a smaller increase of the ESM signal intensity compared to the fresh sample can be obtained (see Figure 4a). Hence, the slope of the ESM signal upon stepwise increasing voltage amplitude is strongly reduced at the aged compared to the fresh cathode. The stepwise increase of the ESM signal intensity for the aged sample is nearly the same for both locations, while the slope differs in the two locations from the fresh sample. This could point towards a homogeneous redistribution of the remaining Li-ions within the aged sample and a levelling of the activity over aging. Additionally, the absolute ESM signal intensity is much smaller compared to the fresh cathode.

The decrease of the ESM signal intensity is more evident in the histograms in Figure 6. Figure 6a shows the ESM signal intensity due to the positive and Figure 6b due to the negative dc-voltage pulse. The histograms are a combination of five measurements from different locations in the cathodes, each with a scan size of 1 µm. Both histograms show a decrease of the ESM signal at higher intensity and an increase of the lower ESM signal intensities from the aged cathode in comparison to the fresh sample. We used the two-sample Kolmogorov–Smirnov test (kstest2 in Matlab R2018) with a significance level of *p* = 0.05 to evaluate if the datasets represent different distributions [59]. Indeed, both datasets passed the test, which indicates a different distribution for the fresh and aged dataset and thereby shows a significant change in the ESM signal due to the ageing. To exclude any tip-related influence on the ESM signal intensity decrease, the AFM tip, which was used to collect the measurements for the fresh sample, was reused for the aged sample to compare the measurements of the reused tip with the measurements of a fresh AFM tip. The reused tip from the fresh sample showed the same ESM signal intensities in the aged sample as for a fresh AFM tip, which verified the tip-independent ESM signal intensity decrease in the aged sample.

Several groups link the ESM signal intensity to the electrochemical activity of Li-ions [33,36,42–44,58,60–61], following the approach applied for the analysis of the piezoresponse force microscopy (PFM), for which the signal amplitude represents the electromechanical activity [62–64]. Similar to the ESM and PFM signal, digital volume correlation (DVC) in combination with in situ X-ray tomography microscopy (XTM) was used by Pietsch et al. [47] for graphite and silicon anodes and by Finegan et al. [48] for a LiMnO_2_ cathode to visualize volume expansion of the electrodes. These results were used to link the local volume expansion of the material to its local activity. Following this assumption, a decrease of the overall ESM signal with ageing would imply a decrease of the electrochemical activity of the Li-ions in the cathode material due to ageing. A decrease of the electrochemical activity of the cathode could result in a smaller current peak intensity in a cyclic voltammetry (CV) experiment. However, performing CV with the fresh and an aged cathode in combination with a fresh graphite anode as counter electrode for both cathodes under test showed a minor decline of the resulting current of the aged cathode and a decrease of the capacity during the anodic and cathodic scans (Supporting Information File 1, Figure S4), which could indicate a reduction of the active phase. However, since not all particles participate simultaneously in the lithiation and delithiation process, the current does not represent the actual, local current density at the particles itself, since only a fraction of the active area is involved [65–66]. Deactivation of particles or loss of active material, which is a known degradation mechanism for LFP cathodes [6,67–68], could be compensated by an increasing local current density at the remaining active particles. Since the CV technique evaluates the entire electrode area, information about local variations are not obtained. However, the reduction of the current and capacity in the CV could support the assumed decrease of the electrochemical activity, suggested by the reduction of the ESM signal intensity. In former studies of LFP degradation, the main effect observed was iron dissolution and Fe^2+^ migration to the anode and redeposition. Fe particles on the anode play a decisive role in accelerated SEI formation [9,69–70]. Iron dissolution from LFP has been found to increase with water content of the electrolyte and phase impurities in the cathode. The dissolution of iron leads to Fe-deficient inactive phases. The aged cathode showed a higher Fe content on the cathode surface and lower Fe content in the cross-section (Fe mass content fresh: 32.1 ± 0.3% and aged: 27.8 ± 0.8%), indicating iron dissolution from the bulk material [71].

Another factor influencing the ESM signal is the structure of the material. Chen et al. observed a dependency of the crystallinity of LFP on the ESM signal and concluded that the nanocrystalline sample must exhibit a higher diffusivity than the comparable microcrystalline structure [44]. We do not expect a change in the overall structure of the sample and therefore neglect this possibility as an influence. Other possible variations of the ESM signal intensity might result from material stiffness or elasticity because these material properties influence the volume expansion. Harder materials are assumed to show a smaller surface displacement (and thus smaller volume expansion) than softer materials. Analysis of the elasticity of the cathode materials was conducted with PeakForce QNM measurements for which the deformation (penetration depth of the tip) of the measurements was evaluated (Supporting Information File 1, Figure S6). The results indicate a constant deformation for the fresh sample, while the aged cathode shows some areas with a higher deformation, indicating a softer material. The change of the material property itself would not influence the decline of the ESM signal intensity over ageing, since the softening would promote higher ESM signal intensities. However, if the softer response represents the Fe-deficient inactive phase as a consequence of iron dissolution the lower ESM signal is a direct consequence. It is noted that this is probable, as Fe-dissolution has been reported as the prominent degradation mechanism of LFP [9,72–73].

**Figure 7:**
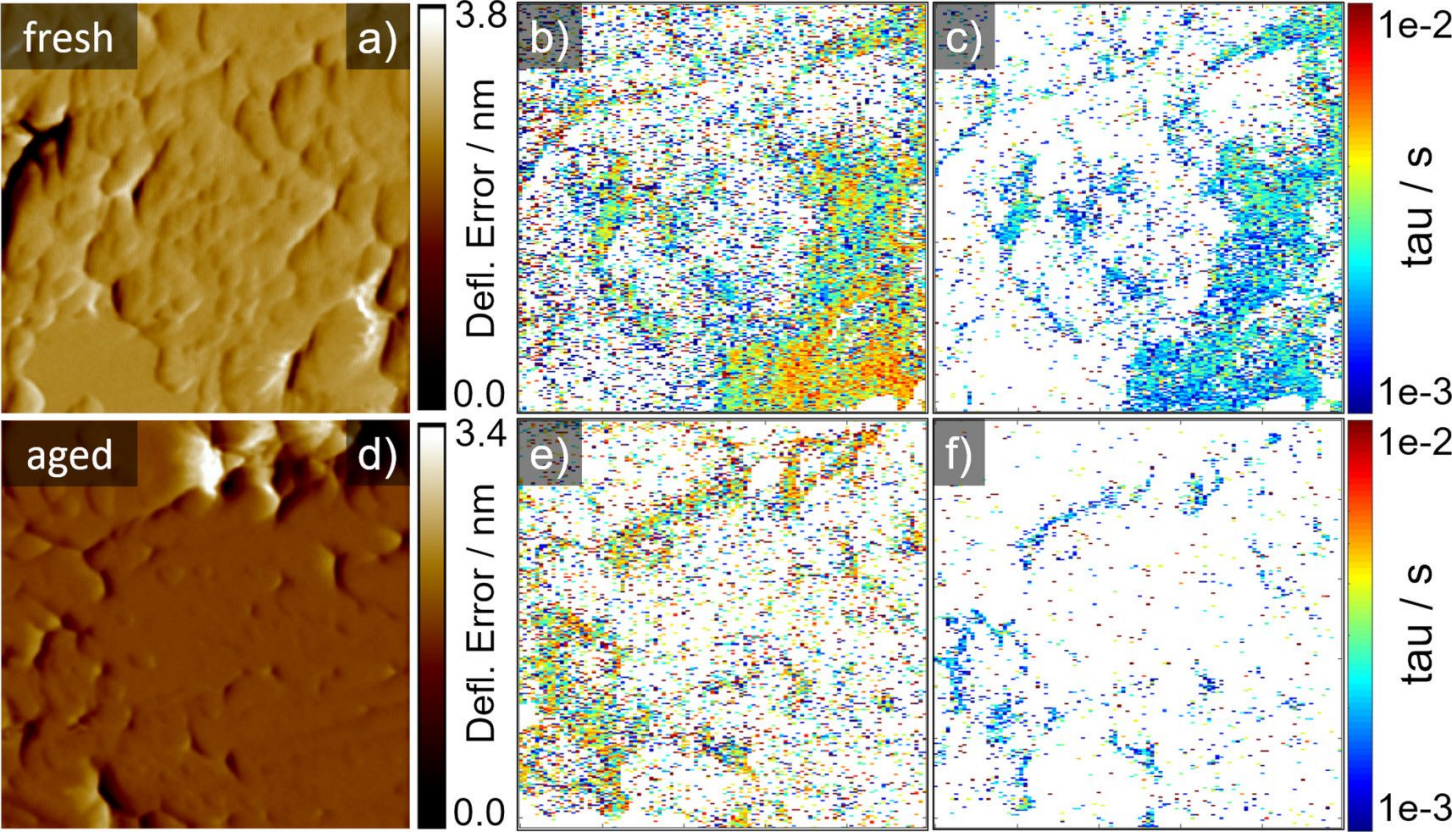
Fitted time constants from Figure 5 of a fresh (top row) and aged cathode (bottom row) cross-section. a) and d) show the deflection error, b) and e) the time constants during positive and c) and f) during negative voltage pulse. Scan size is 1 × 1 µm^2^.

**Figure 8:**
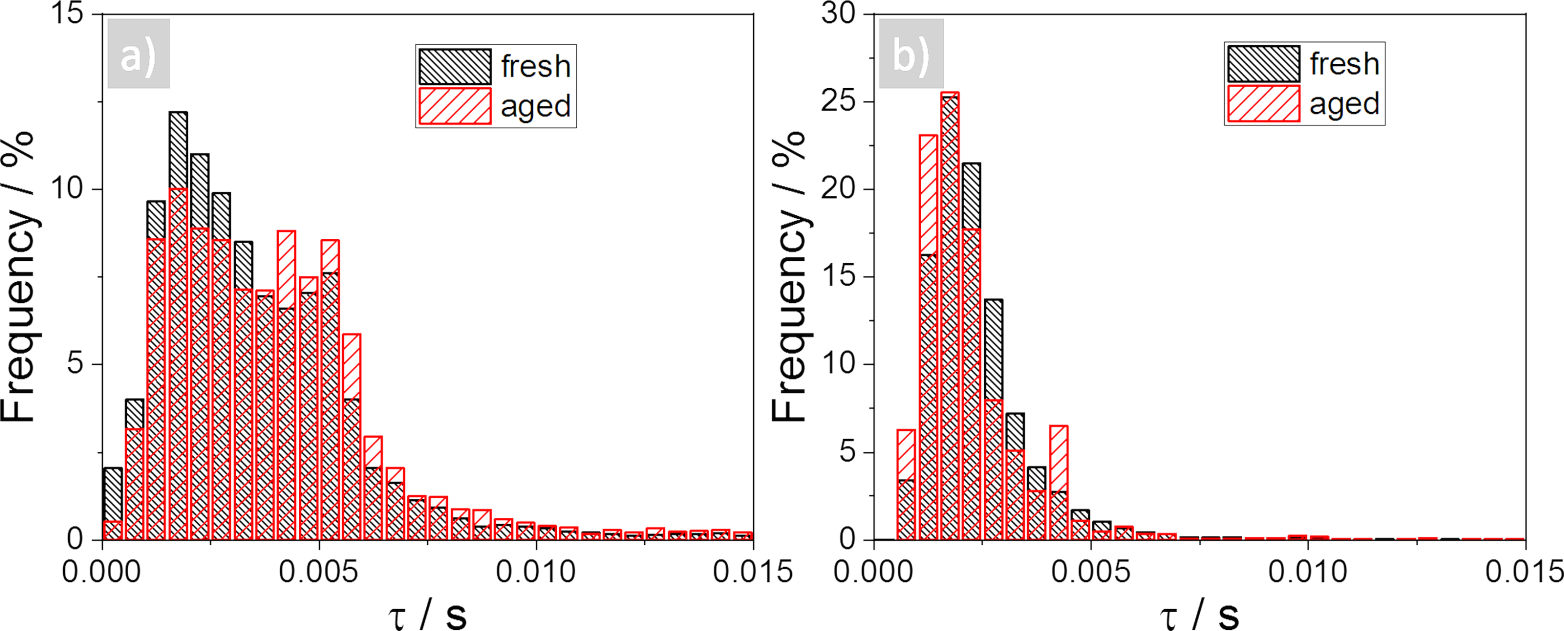
Comparison of time constants of the fresh and aged cathode, a) shows the time constants due to the positive and b) the negative dc-voltage pulse.

The dynamics of the relaxation process after the dc-voltage pulse are further analysed in Figure 7 and Figure 8. Figure 7 shows the same sample location as in Figure 5, but Figure 7b and 7e now present relaxation times after a positive dc-voltage pulse and Figure 7c and 7f after the negative dc-voltage pulse. We excluded data points below a certain threshold, since a minimal ESM signal intensity is needed to generate a fit. Two examples for the resulting fits are given in Supporting Information File 1, Figure S7. The time constants are in the range between 1 and 10 ms, the negative dc-voltage pulse resulting in smaller time constants as compared to the positive pulse. In Figure 8 the relative distribution of the time constants of the fresh and the aged cathode are compared, where Figure 8a shows the positive and Figure 8b the negative dc-voltage pulse. The histograms result from a combination of five different measurements at different locations, the same as for the histograms in Figure 6. Interestingly, the distributions of the positive and negative dc-voltage pulse differ, whereby the negative dc-voltage pulse shows smaller time constants compared with the positive pulse. This indicates a different behaviour of the diffusivity depending on an accumulation (negative pulse) and a depletion (positive pulse) of the Li-ions. The distributions do not show any significant change from the fresh to the aged cathode, indicating an unaffected diffusivity in the material providing the signal. Using 
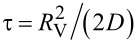
 to describe the diffusion in a thin film [40] with a time constant τ = 2 ms and a depth of the probed volume of roughly *R*_V_ = 10 nm leads to diffusion coefficients of about 2.5 × 10^−14^ m^2^ s^−1^. This value is at the higher bound of experimental values given in the literature which range between 1 × 10^−14^ to 1 × 10^−20^ m^2^ s^−1^ [74–76] and the lower bound of theoretically calculated diffusion coefficients. Theoretical work gives values ranging from 1 × 10^−11^ to 1 × 10^−14^ m^2^ s^−1^, which depends on the direction of the diffusion channels considered [56–57,65,77]. The experimentally generated diffusion coefficients are strongly dependent on the measurement method applied and its analysis (e.g., electrochemical impedance spectroscopy (EIS), potentiostatic intermittent titration technique (PITT), galvanostatic intermittent titration technique (GITT), CV). These techniques assume a simultaneous participation of all LFP particles in the electrodes when a “domino-cascade” is presumed to more accurately reflect the reaction model [56,65,78]. This faulty assumption affects the diffusion coefficients extracted from the experimental data. Additionally, the preparation of the material influences the diffusion coefficient [75,77]. The assumed 10 nm of probed depth is a reasonable assumption considering the sharp boundaries observed in the ESM signals. Smaller values for the probed depth are however possible, which would change the diffusion coefficients by one or two orders of magnitude. Regarding the comparison of fresh and aged diffusion coefficient distributions, the minor differences between the fresh and aged cathodes point towards a stable diffusivity of the cathode material, which is not influenced by any mechanical, electrical or electrochemical degradation. Sun et al. compared the diffusion coefficients of aged and fresh LFP cathodes from half-cell measurements and found only a minor decrease of the diffusion coefficient due to ageing. However, they correlate this to the surface layer build up on the cathodes and not to any degradation of the cathode material itself [6]. Regarding the discrepancy between experimentally and theoretically derived diffusion coefficients, Malik et al. [65] pointed out that the experimentally generated diffusion coefficients with cell level measurements represent the cathode as a whole system and not the bulk properties of LFP particles. For cell level measurements, all particles are assumed to lithiate or delithiate simultaneously. However, this assumption does not hold for a multi-particle system like the electrodes consisting of nanometer- and micrometer-sized particles [65–66]. The assumption leads to an overestimation of the active particle area and therefore to an underestimated diffusion coefficient. The domino-cascade model by Delmas et al. takes into account the coexistence of a lithiated and delithiated phase for LFP [78]. Additionally, other mechanisms and factors, e.g., the generation of surface layers, porosity and tortuosity of electrodes, electrolyte salts and concentration gradients in the electrodes are affecting the ion transport and therefore influence the diffusion coefficients measured on the cell level [79–82]. The formation of an inactive phase by ageing which does not significantly contribute to the ESM signal is consistent with our observation that diffusion coefficients do not change.

Combining the results from the reduction of the ESM signal intensity with the unaffected diffusion coefficient over ageing, the reduced lithium concentration in the aged cathode and the results of the CV from the fresh and aged cathode leads us to the conclusion that the reduction of the ESM signal intensity represents a decrease of the Li-ion concentration inside the probed volume of the aged cathode compared to the fresh cathode and a formation of an inactive phase. A reduced active Li content in the aged cathode reduces the amount of ions, which can be driven by the electric field, which in turn decreases the ionic concentration change and therefore leads to a decrease of the ESM signal intensity. The reduced activity is the result of deactivation of the cathode material, which turned partially electrochemical inactive over ageing. However, since the ionic concentration affects the activity as well, it is difficult to clearly state the main source for the reduced ESM signal intensity [83]. If the ESM signal would only represent the mobility of the Li-ions, the reduction of the ESM signal intensity would be visible in the decrease of the diffusion coefficient, which is not observed.

## Conclusion

This paper presents a tailored ESM technique, which is used to study the ageing of LFP cathodes. First, a fresh cathode cross-section is analysed. The measurements show a higher ESM signal intensity at structural borders within single grains, but active locations at homogenous and planar areas as well. The activity and the ionic concentration in the material influence the ESM signal. Using ESM voltage spectroscopy, a linear increase of the ESM signal is observed for the fresh cathode with no visible side reactions on the cross-section surface. Comparison with the ESM signal at the cross-section of an aged cathode reveals a distinctly different behaviour. There is a smaller amount of ESM active area on the cross-section surface and the overall, absolute signal intensity is smaller compared to the fresh cathode. ESM voltage spectroscopy indicates, as it was observed for the fresh cathode, a linear dependency of the ESM signal with the voltage amplitude. However, the slope of the ESM signal is much smaller for the aged cathode, as compared to the fresh one. Fitting the ESM relaxation after the applied voltage pulse provides time constants which represent diffusion coefficients in the range of 2.5 × 10^−14^ m^2^ s^−1^. These values are in the range of theoretical and experimental values found in the literature. Combining all the analysis leads to the conclusion that a reduction in the electrochemical activity and Li content in the cathode is responsible for the reduction of the ESM signal intensity most probably by formation on an inactive phase. Both mechanisms likewise influence the remaining cathode capacity, which is reduced due to the ageing.

## Supporting Information

File 1 Further experimental data of cell and electrode characterisation.
