# Supplementary material for: Comparison of fresh and aged lithium iron phosphate cathodes using a tailored electrochemical strain microscopy technique
Source: Beilstein J Nanotechnol. 2020 Apr 7;11:583–96. doi: 10.3762/bjnano.11.46 (PMC7155899; doi:10.3762/bjnano.11.46)
Supplement: File 1 — Further experimental data of cell and electrode characterisation. [file Beilstein_J_Nanotechnol-11-583-s001.pdf]

BEILSTEIN JOURNAL OF NANOTECHNOLOGY

Supporting Information

for

Comparison of fresh and aged lithium iron phosphate cathodes using a tailored electrochemical strain microscopy technique

Matthias Simolka, Hanno Kaess and Kaspar Andreas Friedrich

Beilstein J. Nanotechnol. **2020,** *11,* 583–596. doi:10.3762/bjnano.11.46

Further experimental data of cell and electrode characterisation

License and Terms: This is a supporting information file under the terms of the Creative Commons Attribution License (http://creativecommons.org/licenses/by/4.0). Please note that the reuse, redistribution and reproduction in particular requires that the authors and source are credited.
The license is subject to the *Beilstein Journal of Nanotechnology* terms and conditions: (https://www.beilstein-journals.org/bjnano)

The discussion about non-Vegard contributions is taken from our supporting information section of our previous paper [1] and adapted to LFP:

Non-Vegard contributions to the ESM signal

Other mechanisms that can contribute are i) inverse piezoelectric effect, flexoelelectric effect and electrostriction ii) deformation potential, electron-hole and electron-phonon coupling, iii) electrochemical reactions on the surface, iv) electrostatic influence and electric Lorenz-like forces and v) temperature related volume expansion

Piezoelectricity, flexoelectricity and electrostriction are assumed not to be the primary mechanism for the signal generation, due to measurements on fresh silicon, Si/C anode, carbon, HOPG and polyether ether keton (PEEK), which did not show a signal generation dependency on the applied voltage profile. Nevertheless, since ions are influencing these effects, especially electrostriction, it is difficult to rule them out completely in the lithiated samples. However, Kalinin and Morozovska are reporting their influence to be at least one order of magnitude smaller, compared to the Vegard expansion [2].

The deformation potential and electron-hole and electron-phonon coupling are expected to contribute only by one order of magnitude smaller compared to Vegard expansion [2–5].

Surface reactions with impurities or surface layers can lead to tip-surface expansion. Surface reactions usually alter the sample surface or the AFM tip and are therefore detected by surface changes and loss in resolution. However, surface reactions can occur without surface changes. In this case, the reaction needs an electrolyte, such as water, to serve as a product and educt reservoir. Since the measurements are carried out in an argon filled glovebox, the appearance of a water meniscus at the tip-sample junction can be excluded.

Trapped charges in the probed material can influence the signal. Additionally, charges can be injected by the AFM tip. However, charge injection from the AFM tip is unlikely, since only small voltage amplitudes are used, but cannot be ruled out completely. Trapped charges and charge injection would have occurred at the fresh samples during the control experiments, which were not observed. The influence of surface and space charges can be evaluated using the Maxwell-Wagner relaxation time. Using typical values for the dielectric permittivity of LFP (between 5 and 12 [6]) and the electrical conductivity of around 10^{-6} S/m [7], the relaxation time ($\tau = \frac{\varepsilon\varepsilon_0}{4\pi\sigma}$, with $\varepsilon_0 = 8.85 \cdot 10^{-12}\,\mathrm{As\,V^{-1}m^{-1}}$ leading to roughly 10^{-6} s and lower) is several orders of magnitude smaller than the measured signal relaxation time (around 10^{-3} – 10^{-1} s) and values found in the literature [8,9].

Some authors assume an electronic Lorenz-like force acting on the ions in the sample as the primary source of the ESM signal. The ions do not hop from lattice site to another lattice site, which would lead to the Vegard expansion, but vibrate on the lattice site, due to the alternating electric field [10–12].

The application of electric potentials between the AFM tip and the sample surface can generate heat due to energy dissipation, since the tip-sample junction acts as a resistor. For a primary estimation of the temperature influence, we will make some simplified calculations. The dissipated energy Q, as well called Joule heating, is given in the simplest form:

$$Q = \frac{\Delta\phi}{R_{t-s}^2} \tag{S.1}$$

With $\Delta\phi$ as the electrical potential difference between the tip and the sample and R_{t-s}^2 as the contact resistance between tip and sample surface [13,14]. With the energy Q given and limiting the energy dissipation to the change of the temperature of the

sample surface ΔT_s and a small, cubic volume element of the sample surface under the tip V_s, the change in temperature is given by :

$$\Delta T_s = \frac{Q}{V_s\, \rho_s\, c_p} \qquad \text{(S.2)}$$

With ρ_s as the density of volume element of the sample surface, which is influenced by the heating, and c_p as the heat capacity of the sample surface at constant pressure [15]. The change in temperature follows a strain ε_T^A or deformation u_T^A of the sample surface:

$$\varepsilon_T^A = \alpha_S\, \Delta T_s \qquad \text{(S.3)}$$

$$u_T^A = \alpha_S\, \Delta T_s\, l_0^{V_s} \qquad \text{(S.4)}$$

Here, α_S is the thermal expansion coefficient of the sample surface and $l_0^{V_s}$ is the vertical length of the volume element [16,17]. Assuming a contact resistance of 100 MOhm between a conductive tip and the sample , a potential difference of 10 V, a volume of $1 \cdot 10^{-21}$ m^3 of the sample to be influence by the heating, the density of LFP with 3600 kg m^{-3}, a specific heat capacity of LFP of roughly 800 J kg^{-1} K^{-1} [18] and a thermal expansion coefficient of LFP with $5.3 \cdot 10^{-5}$ K^{-1} [19], the thermal deformation is in the range of about 10 fm and therefore at least two orders of magnitude smaller than the measured signal intensity. [20–22]. Here we assumed for simplification, that all the dissipation energy is transferred into the sample. The surrounding media as well as the tip will absorb part of the dissipated energy, which will decrease the temperature change of the sample and the volume expansion.

Regarding all the considered other mechanisms, we still assume the signal to have an ionic origin with the Vegard expansion being the main mechanism responsible for the surface displacement.

Characterization of the cathodes in commercial 26650 cells and three-electrode test cell

Commercial 26650 $LiFePO_4$ cells from A123 Systems LLC are used. Full cell and three-electrode test cell measurements are conducted using a battery cycler (CTS-XL, BaSyTec GmbH) with a two-electrode and three-electrode configuration. Three-electrode test cell measurements are conducted using electrodes with 18 mm in diameter, which are punched out of the fresh and aged electrode foils from the commercial cells., A PP/PE membrane (FS-5P, EL-CELL GmbH) was used as separator. For the three-electrode tests, the electrolyte was 1 Mol $LiPF_6$ in EC/DMC (1:1 vol%, Sigma Aldrich). The three-electrode tests were conducted using a commercial test cell (PAT-Core, EL-CELL GmbH) with a lithium metal ring as reference electrode. One side of the electrode material was removed from the current collector using N-Methyl-2-pyrrolidone (NMP, Sigma Aldrich). The three-electrode test cells were assembled using a fresh or aged cathode in combination with a fresh anode from the uncycled full cell to avoid any ageing influence from the anode side. All work was conducted in an argon filled glovebox.

Discharging profile from fresh and aged Commercial 26650 cells

In Figure S1, the 1C discharge curves for a commercial fresh and aged cell are presented. A capacity loss of the aged cell after end of ageing of 17% is observed.

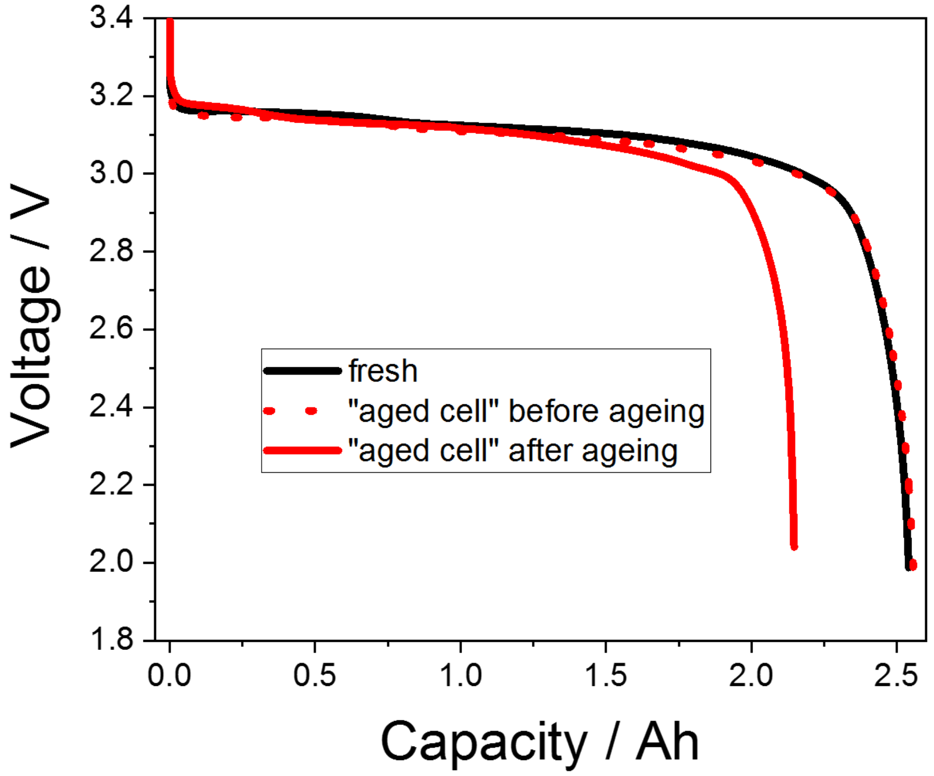

Figure S1: Voltage over capacity of the fresh and aged full cell

Electrochemical impedance spectroscopy measurements from the three-electrode test cells from the fresh and aged cathode vs. lithium metal

Impedance measurements are conducted using a Princeton Applied Research Versastat 450 potentiostat with an AC amplitude of 10 mV in the frequency range from 500 kHz to 0.5 mHz at OCV.

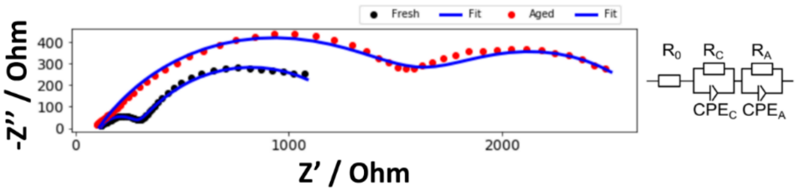

Figure S2: Nyquist plot of the fresh (black) and aged (red) cathode vs. a lithium reference ring electrode in discharged state

$R_{C,\ fresh}$ = 172 Ω $R_{A,\ fresh}$ = 1060 Ω

$R_{C,\ aged}$ = 1140 Ω $R_{A,\ aged}$ = 1530 Ω

Impedance data is analyzed using the impedance.py python package. [23]

First charge and discharge step from three-electrode test cells

Figure S3 displays the first charge and discharge step from the three-electrode test cells with a fresh and aged cathode, both with a fresh anode. The voltage curve represents the potential between cathode and anode. We used 0.185 mA as charge and discharge current, since that represents roughly C/20, and therefore provide the maximum of the accessible capacity. The cell was first charged up to 3.6 V with a constant voltage step until the current dropped below 0.120 mA and afterwards discharged to 2.0 V.

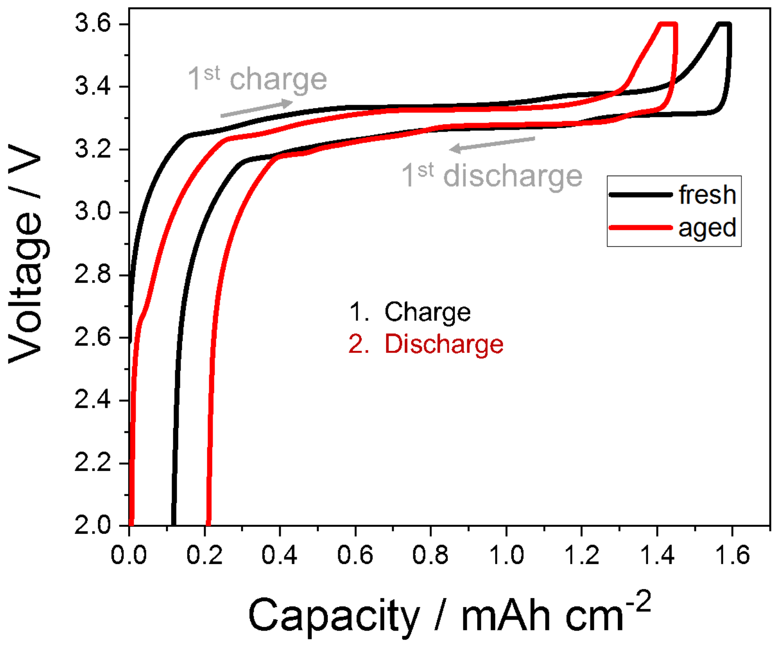

Figure S3: Full-cell test of the fresh and aged cathode combined with a fresh anode

Cyclic voltammetry (CV) measurements of the fresh and aged cathode in the three-electrode setup

For the CV, the three-electrode setup with a fresh or aged cathode in combination with a fresh anode and a lithium metal reference ring was used. The scan rate was set to 1 mV s^{-1} in the range from 2.0 V to 3.6 V regarding the potential between cathode and anode.

The area under the CV peaks or the available capacity during the anodic scan for the fresh cathode – fresh anode combination is 4.6 mAh and for the aged cathode – fresh anode combination only 4.0 mAh. The capacity during the cathodic scan for the fresh cathode – fresh anode combination is 4.7 mAh and for the aged cathode – fresh anode combination only 4.0 mAh.

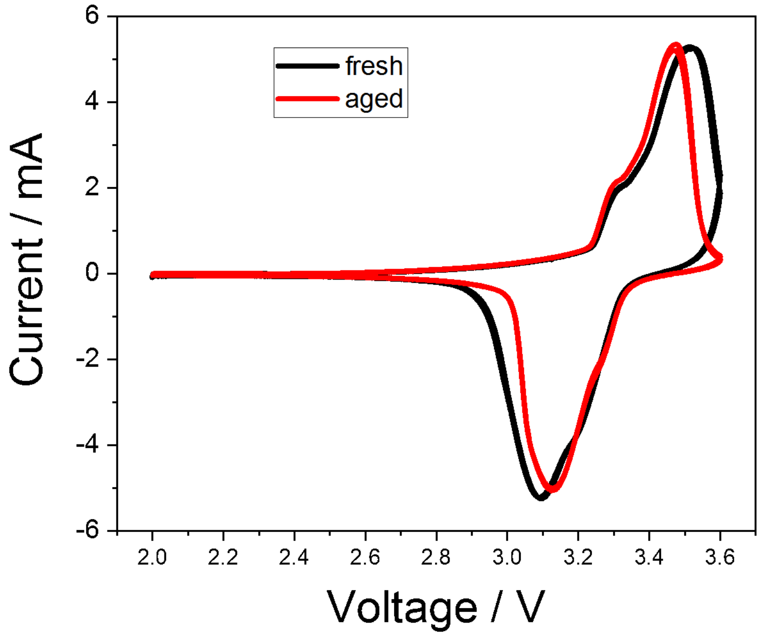

Figure S4: CV of the full-cell test setup with the fresh (black) and aged (red) cathode combined with a fresh anode. The potential is measured between cathode and anode.

ESM measurements of the aged cathode with stepwise increasing dc-voltage amplitude

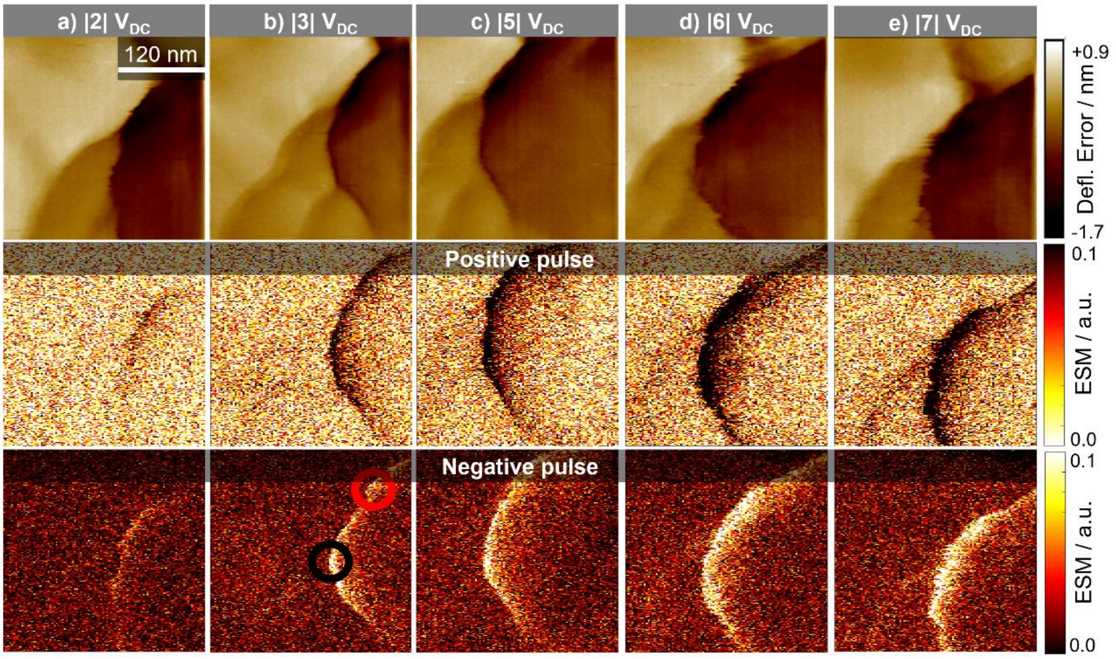

Figure S5: Aged cathode, comparison of different dc-voltage amplitudes at the same location. The top row shows the deflection error, the middle row the ESM signal during positive and the bottom row during negative dc-voltage pulse. In a) with |2|V, b) with |3|V, c) with |5|V, d) with |6|V and e) with |7|V. Scan size is 0.33 µm.

Deformation of the fresh and aged cathodes measured with Bruker PeakForce Quantitative Nanomechanical Properties mode (QNM)

For the fresh and aged sample, a total area of 32 µm² was analysed, using the same settings and the same tip.

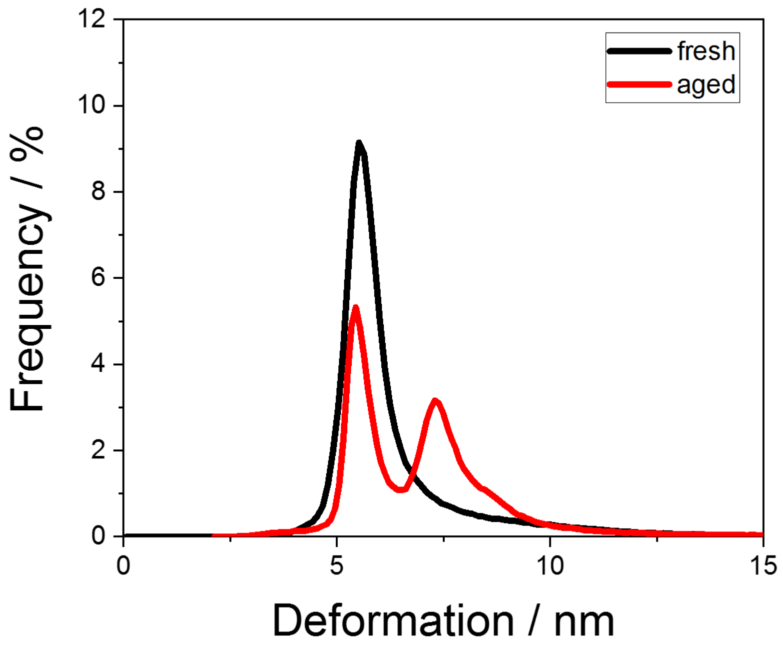

Figure S6: Comparison of the deformation distribution of the fresh and aged cathode cross-section

Examples of the fitting results for the relaxation process

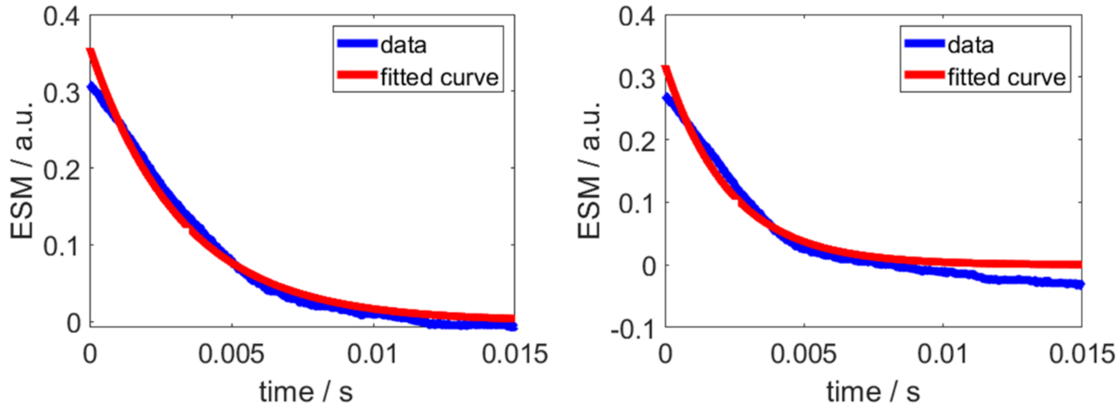

Figure S7: Fitting examples for the relaxation ESM signal

The mean RMSE is in the order of 0.013.

References

[1] Simolka, M.; Heim, C.; Friedrich, K. A.; Hiesgen, R. *J. Electrochem. Soc.* **2019**, *166* (3), A5496–A5502. https://doi.org/10.1149/2.0711903jes.

[2] Kalinin, S. V.; Morozovska, A. N. *Journal of Electroceramics* **2014**, *32* (1), 51–59. https://doi.org/10.1007/s10832-013-9819-7.

[3] Kleinman, L. *Physical Review* **1962**, *128* (6), 2614–2621. https://doi.org/10.1103/PhysRev.128.2614.

[4] Fischetti, M. V.; Laux, S. E. *Journal of Applied Physics* **1996**, *80* (4), 2234–2252. https://doi.org/10.1063/1.363052.

[5] Morozovska, A. N.; Eliseev, E. A.; Tagantsev, A. K.; Bravina, S. L.; Chen, L.-Q.; Kalinin, S. V. *Physical Review B* **2011**, *83* (19). https://doi.org/10.1103/PhysRevB.83.195313.

[6] Islam, M. S.; Driscoll, D. J.; Fisher, C. A. J.; Slater, P. R. *Chem. Mater.* **2005**, *17* (20), 5085–5092. https://doi.org/10.1021/cm050999v.

[7] Johnson, I. D.; Lübke, M.; Wu, O. Y.; Makwana, N. M.; Smales, G. J.; Islam, H. U.; Dedigama, R. Y.; Gruar, R. I.; Tighe, C. J.; Scanlon, D. O.; et al. *Journal of Power Sources* **2016**, *302*, 410–418. https://doi.org/10.1016/j.jpowsour.2015.10.068.

[8] Jesse, S.; Balke, N.; Eliseev, E.; Tselev, A.; Dudney, N. J.; Morozovska, A. N.; Kalinin, S. V. *ACS Nano* **2011**, *5* (12), 9682–9695. https://doi.org/10.1021/nn203141g.

[9] Guo, S.; Jesse, S.; Kalnaus, S.; Balke, N.; Daniel, C.; Kalinin, S. V. *Journal of the Electrochemical Society* **2011**, *158* (8), A982–A990.

[10] Amanieu, H.-Y.; Thai, H. N. M.; Luchkin, S. Yu.; Rosato, D.; Lupascu, D. C.; Keip, M.-A.; Schröder, J.; Kholkin, A. L. *Journal of Applied Physics* **2015**, *118* (5), 055101. https://doi.org/10.1063/1.4927747.

[11] Schön, N.; Gunduz, D. C.; Yu, S.; Tempel, H.; Schierholz, R.; Hausen, F. *Beilstein Journal of Nanotechnology* **2018**, *9*, 1564–1572. https://doi.org/10.3762/bjnano.9.148.
[12] Lushta, V.; Bradler, S.; Roling, B.; Schirmeisen, A. *Journal of Applied Physics* **2017**, *121* (22), 224302. https://doi.org/10.1063/1.4984831.
[13] Ansari, M. Z.; Cho, C. *Sensors* **2010**, *10* (11), 9668–9686. https://doi.org/10.3390/s101109668.
[14] Cole, K. D.; Çetin, B. *International Journal of Thermal Sciences* **2017**, *119*, 24–36. https://doi.org/10.1016/j.ijthermalsci.2017.05.010.
[15] Tilley, R. J. D. John Wiley & Sons, Ltd*Understanding Solids: The Science of Materials*, 2004.
[16] Landau, L. D.; Lifshitz, E. M. **1970**, 177.
[17] Czichos, H.; Skrotzki, B.; Simon, F.-G. Springer Berlin Heidelberg: Berlin, Heidelberg*Das Ingenieurwissen: Werkstoffe*, 2014. https://doi.org/10.1007/978-3-642-41126-7.
[18] Nanda, J.; Martha, S. K.; Porter, W. D.; Wang, H.; Dudney, N. J.; Radin, M. D.; Siegel, D. J. *Journal of Power Sources* **2014**, *251*, 8–13. https://doi.org/10.1016/j.jpowsour.2013.11.022.
[19] Katoh, Y., Fox, K. M., Lin, H.-T., Belharouak, I., Eds.; John Wiley & Sons, Ltd*Ceramic Materials for Energy Applications*, 2011.
[20] Tsukruk, V. V.; Singamaneni, S. Wiley*Scanning Probe Microscopy of Soft Matter: Fundamentals and Practices*, 2012.
[21] Chowdhury, F. K.; Pourzand, H.; Tabib-Azar, M. 4.
[22] De Wolf, P.; Snauwaert, J.; Clarysse, T.; Vandervorst, W.; Hellemans, L. *Applied Physics Letters* **1995**, *66* (12), 1530–1532. https://doi.org/10.1063/1.113636.
[23] ECS Hack Week *A Python Package for Working with Impedance Data. Contribute to ECSHackWeek/Impedance.Py Development by Creating an Account on GitHub*, 2019.
